# Natural killer cell engagers for cancer immunotherapy

**DOI:** 10.3389/fonc.2024.1483884

**Published:** 2025-01-22

**Authors:** Shahryar Khoshtinat Nikkhoi, Geng Li, Arash Hatefi

**Affiliations:** ^1^ Department of Pharmaceutics, Rutgers University, Piscataway, NJ, United States; ^2^ Cancer Pharmacology Program, Cancer Institute of New Jersey, New Brunswick, NJ, United States

**Keywords:** NKCE platform technologies, NK activating receptors, bispecific trispecific multispecific antibodies, CD16a NKG2D NKG2C NKp30 NKp46, cancer immunotherapy, natural killer cells (NK cells)

## Abstract

This review article explores the rapidly evolving field of bi-, tri-, and multi-specific NK cell engagers (NKCEs), highlighting their potential as a cutting-edge approach in cancer immunotherapy. NKCEs offer a significant advancement over conventional monoclonal antibodies (mAbs) by enhancing Antibody-Dependent Cellular Cytotoxicity (ADCC). They achieve this by stably and selectively binding to both NK cell activating receptors and tumor-associated antigens (TAAs). Unlike traditional mAbs, which depend on the relatively transient interaction between their Fc region and CD16a, NKCEs establish more robust connections with a range of activating receptors (e.g., CD16a, NKG2D, NKp30, NKp46, NKG2C) and inhibitory receptors (e.g., Siglec-7) on NK cells, thereby increasing cancer cell killing efficacy and specificity. This review article critically examines the strategies for engineering bi-, tri-, and multi-specific NKCEs for cancer immunotherapy, providing an in-depth analysis of the latest advancements in NKCE platform technologies currently under development by pharmaceutical and biotech companies and discussing the preclinical and clinical progress of these products. While NKCEs show great promise, the review underscores the need for continued research to optimize their therapeutic efficacy and to overcome obstacles related to NK cell functionality in cancer patients. Ultimately, this article presents an overview of the current landscape and future prospects of NKCE-based cancer immunotherapy, emphasizing its potential to revolutionize cancer treatment.

## 1 Introduction

Antibody-mediated cancer immunotherapy is a type of treatment approach that harnesses the power of antibodies to bolster the immune system’s ability to fight cancer. These therapeutic antibodies are engineered to recognize and bind to specific antigens present on the surface of cancer cells. Upon binding to these antigens, initiate a series of immune responses, which directs the immune system to target and eliminate the cancer cells (1, 2). Structurally, antibodies are composed of two distinct regions that are integral to their function: the Fab and Fc regions (3). The Fab (fragment antigen-binding) region represents the variable portion of the antibody, which is responsible for recognizing and binding to specific antigens. This region comprises the two arms of the Y-shaped antibody molecule and contains the antigen-binding sites. In contrast, the Fc (fragment crystallizable) region is the constant portion of the antibody that mediates interactions with various components of the immune system (4). The Fc region determines the isotype of the antibody and its functional properties, including its capacity to bind to Fc gamma receptors (FcγRs) found on immune cells like natural killer (NK) cells, T cells, macrophages, neutrophils, and B cells (5, 6). In humans, FcγRs include several activating receptors, such as FcγRI (CD64), FcγRIIa (CD32a), FcγRIIc (CD32c), FcγRIIIa (CD16a), and FcγRIIIb (CD16b), along with the inhibitory receptor FcγRIIb (CD32b), which contains an immunoreceptor tyrosine-based inhibitory motif (ITIM) (7). The expression and activity of these receptors are tightly regulated by factors such as cytokines, cell type, and activation state. For example, FcγRI expression is upregulated in response to interferon-gamma (IFN-γ), while FcγRIIb expression is modulated by signaling through the B cell receptor and other co-stimulatory pathways (8, 9). In tumors, FcγR expression patterns are often altered; tumor-associated macrophages frequently exhibit increased FcγRI and decreased FcγRIIb expression, shifting the balance toward an activating phenotype (10). Activating FcγRs possess immunoreceptor tyrosine-based activation motifs (ITAMs) that initiate downstream signaling cascades, leading to effector functions such as antibody-dependent cellular cytotoxicity (ADCC), phagocytosis, and pro-inflammatory cytokine production. Different IgG subclasses bind to FcγRs with varying affinities; for instance, FcγRI has high affinity for monomeric IgG, whereas FcγRIII preferentially binds IgG1 and IgG3 in immune complexes (11, 12).

NK cells are a critical subset of lymphocytes that play an essential role in the immune system’s defense against tumors and viral infections (13). They express several activating receptors, including CD16a, NKG2D, NKG2C, NKp46, and NKp30, as well as the inhibitory receptor Siglec-7. The CD16a receptor (FcγRIIIa) on NK cells is central to the ADCC mechanism, as it binds to the Fc region of IgG antibodies attached to the target cells. The clinical importance of CD16a interaction with antibody Fc region is highlighted by the success of several FDA-approved cancer therapies, including rituximab (14), trastuzumab (15), and cetuximab (16), all of which rely on CD16a receptor activation to achieve their anti-tumor effects (6). However, upon activation, CD16a can be shed from the cell surface through proteolytic cleavage by ADAM17, providing a mechanism to modulate NK cell activity and prevent excessive immune responses (17).

NKG2D is another activating receptor on NK cells that binds MICA/B and ULBP1-6 ligands, activating NK cells through DAP10 adaptor proteins and promoting cell cytotoxicity. Similarly, the NKG2C receptor pairs with CD94 and signals via DAP12, especially in response to cytomegalovirus (CMV) infection, enhancing NK cell activity and counteracting inhibitory signals from NKG2A. NKp46 is another interesting activating receptor on NK Cells which associates with CD3ζ or FcRγ to trigger cytolytic functions, while NKp30 induces NK cell activation through its isoforms when interacting with ligands such as B7-H6. Upon forming an immunological synapse with target cells, NK cells degranulate, releasing cytotoxic molecules like perforin and granzyme B, which induce lysis in target cells (18). Perforin creates pores in the target cell membrane, facilitating the entry of granzymes and granulysin, which activate apoptotic pathways leading to the destruction of the target cell (19). In contrast to these activating receptors, Siglec-7 is an inhibitory receptor that modulates NK cell activity by binding ligands and signaling through an ITIM motif, thus balancing NK cell function.

Despite the significant promise of ADCC-mediated therapies, several challenges must be addressed to fully harness their therapeutic potential. The efficacy of ADCC can be influenced by multiple factors, including the density of target antigen expression on cancer cells, the affinity and specificity of the antibodies employed, and the presence of inhibitory receptors on immune cells that can dampen the cytotoxic response. Furthermore, the expression and functionality of Fc receptors, such as CD16a, can be modulated by various factors, including cytokines and genetic polymorphisms, which may affect the overall therapeutic outcome (20, 21). Recent advancements have spurred a growing interest in developing strategies to enhance ADCC activity for the treatment of cancer and other diseases (22). One approach involves engineering the Fc region of therapeutic antibodies to improve their binding affinity for CD16a, thereby augmenting ADCC activity (23, 24). For instance, margetuximab, an Fc-engineered version of trastuzumab, has been modified to enhance its interaction with CD16a, leading to improved ADCC efficacy as demonstrated by both preclinical and clinical studies (25). However, a notable challenge with this strategy is the potential for increased interaction with other Fc receptors, which may lead to unintended effects. For example, engineering of the Fc domain in trastuzumab to create margetuximab resulted in enhanced CD16a (F176) binding affinity (~99 nM, a ten-fold improvement over trastuzumab’s ~1066 nM affinity), although this modification also increased affinity for the inhibitory receptor CD32b on B cells from ~33 nm to ~17 nM, potentially dampening B cell responses (26, 27).

An alternative strategy to improve ADCC efficiency involves using NK cell engagers (NKCEs) (28). NKCE therapies leverage the activating receptors to selectively direct NK cells towards tumor cells, amplifying the immune response by directing the NK cells to tumor-associated antigens (TAAs). By engaging multiple receptors simultaneously, NKCEs boost NK cell activation, helping to overcome immune evasion within the tumor microenvironment and representing a promising approach for cancer immunotherapy. Unlike the transient and relatively low-affinity interaction between the Fc domain of monoclonal antibodies (mAbs) and CD16a, NKCEs could form a stable connection with any activating receptor on NK cells, significantly enhancing ADCC efficiency (24, 29). Moreover, NKCEs are typically engineered to be highly selective and specific to activating receptors on NK cells, minimizing unwanted interactions with other receptors (30). There is considerable ongoing research focused on the development of novel bi-, tri-, and multi-specific NKCEs for NK cell-based cancer immunotherapy. While CD16a has traditionally been the receptor of choice for activating NK cells in antibody therapies, emerging studies suggest that NKCEs targeting other activating receptors can also effectively elicit NK cell responses to kill target cells. In this review, we explore various strategies for developing bi-, tri-, and multi-specific antibodies that engage NK cell surface receptors in cancer immunotherapy. We focus on NKCE platform technologies currently under development by pharmaceutical and biotech companies, discussing the preclinical and clinical progress of these products. The coverage of the literature is not encyclopedic; rather, select examples have been chosen to highlight certain important points. Finally, we explore potential strategies to further enhance the efficacy of NK cell engagers, offering insights into future directions in this rapidly evolving field.

## 2 NK cell engagers

**Figure 1:**
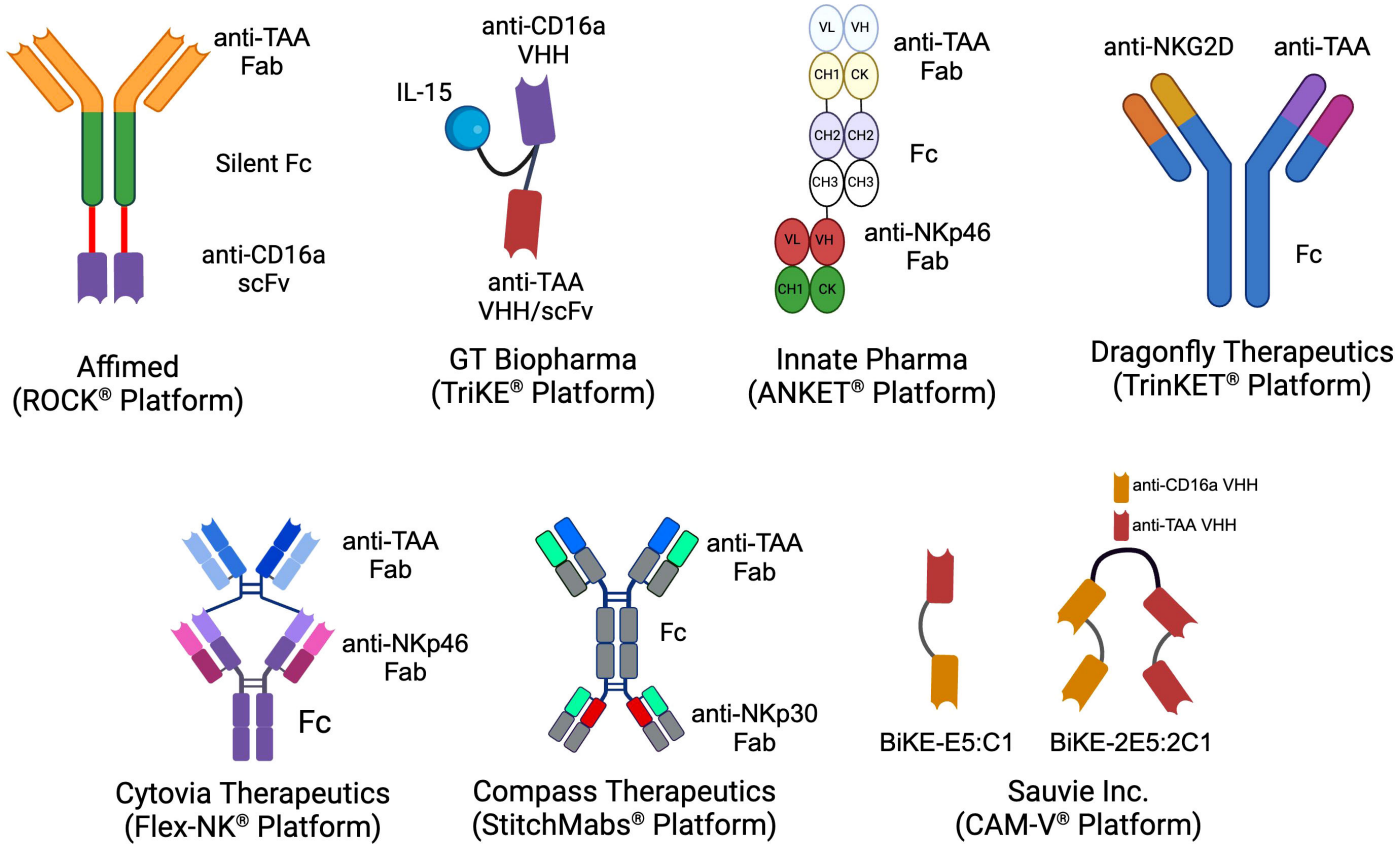
The schematics of the different NKCE platforms currently under development by biotech companies for cancer immunotherapy. (Created with BioRender.com).

In both peripheral and cord blood, NK cells are historically classified into two main subsets based on the expression of CD56 and CD16a: CD56^bright^ CD16a^¯^ and CD56^dim^ CD16a^+^. The CD56^dim^ CD16a^+^ NK cells, which constitute approximately 90% of the NK cell population in the blood, are primarily responsible for executing innate anti-cancer effector functions (31, 32). In contrast, the CD56^bright^ CD16a^¯^ NK cells, making up the remaining 10% of NK cells, are more specialized in cytokine release and exerting regulatory functions (32). The principal mechanism by which NK cells eliminate target cells via NK cell engagers is ADCC, predominantly mediated by theCD56^dim^ CD16a^+^ NK cells (33). Various biotechnology companies have developed NKCEs that target activating receptors on NK cells other than CD16a receptors (**Figure 1**). For example, NKCEs can engage natural cytotoxicity receptors, including NKp30 and NKp46, to trigger NK cell effector functions. The subsequent sections will provide a detailed discussion of these platform technologies and their implications for cancer therapy.

### 2.1 scFv-based NKCEs targeting CD16a receptors

CD16a is an activating receptor comprising extracellular, transmembrane, and intracellular domains, each playing a critical role in its function. The extracellular portion contains two immunoglobulin-like domains, D1 and D2, which are responsible for binding to the Fc region of antibodies (34). The transmembrane domain facilitates connections with intracellular signaling molecules through polar and aromatic residues (35). The intracellular domain interacts with homodimers and heterodimers of CD3ζ (CD247) and/or FcϵR1γ, initiating and transducing activation signals within NK cells, ultimately leading to the full activation of NK cell effector functions (36). The high-affinity activation of CD16a receptors is crucial in cancer immunotherapy because it not only activates resting NK cells (37), but also helps them overcome inhibitory signals from HLA-E, which is often overexpressed on cancer cells (38). Consequently, many NKCEs under development are designed to target CD16a receptors.

For example, Uwe Reusch and colleagues (2014) successfully isolated a CD16a-specific single-chain variable fragment (scFv) through phage display and affinity maturation technologies. This engineered anti-CD16a scFv (code-named LSIV21) was later utilized by Affimed and incorporated into a tetravalent bispecific construct targeting CD30+ tumor cells. The resulting bispecific killer cell engager (BiKE), known as AFM13, demonstrated high affinity for CD16a without binding to CD16b-NA1 or CD16b-NA2 on neutrophils (39, 40). AFM13 exhibited superior potency in killing CD30+ cancer cells compared to both non-engineered and Fc-enhanced anti-CD30 mAbs (40). The anti-tumor efficacy of AFM13 was further enhanced when used in combination with peripheral or cord blood NK cells pre-conditioned with a cytokine cocktail of IL-12, IL-15, and IL-18 (41). Given its high efficacy in preclinical studies, AFM13 advanced to clinical trials. To date, seven clinical trials related to AFM13 (NCT03192202, NCT01221571, NCT02665650, NCT02321592, NCT04101331, NCT04074746, NCT05883449) have been registered, with three completed and results disclosed. AFM13 has been clinically evaluated as both a standalone treatment and in combination with other therapies in patients with CD30+ relapsed or refractory Hodgkin or non-Hodgkin lymphoma, including those who have previously undergone chemotherapy and/or autologous stem cell transplantation and have shown resistance to their most recent therapy (42–44). Although monotherapy with AFM13 yielded modest results (42, 43), its combination with the anti-PD-1 antibody pembrolizumab showed remarkable efficacy, with an overall response rate (ORR) of 83% at the highest evaluated dose (44). This was a significant improvement over monotherapy with pembrolizumab resulting in ORR of 69% as reported in Phase 2 clinical trial for relapsed or refractory Hodgkin lymphoma (45).

In another trial (NCT04074746), the effectiveness of AFM13 combined with cord blood NK cells was evaluated. In this study, AFM13 and cord blood NK cells were first mixed ex vivo and then freshly infused into patients (46). This approach resulted in an ORR of 92.8% and a complete response (CR) rate of 66.7% in patients resistant to CD30-directed brentuximab vedotin treatment (46). Later, Pharmacokinetic (PK) analysis revealed that AFM13 exhibited slightly greater than dose-proportional systemic exposure, with a half-life ranging from 9 to 19 hours (43). Anti-drug antibodies (ADA) and neutralizing antibodies (NAb) against AFM13 were detectable (43, 44), likely due to the murine-derived anti-CD30 sequence (scFv) within the AFM13 construct. Biomarker analysis of patients’ serum showed a reduction in soluble CD30 (sCD30) levels, indicative of AFM13-mediated CD30+ tumor cell lysis (43, 44). In addition to the changes in sCD30 serum levels, variations in the number and phenotype of NK cells in peripheral blood also served as pharmacodynamic biomarkers during therapy. Following AFM13 administration, a decrease in peripheral blood NK cell count was observed, likely due to AFM13-mediated NK cell redistribution (43). The authors describe the NK cell redistribution as the movement and redirection of NK cells from patient’s peripheral blood to the tumor sites following the administration of AFM13. Concurrently, an increase in the expression of CD69, a marker of NK cell activation, was observed on patients’ NK cells immediately after AFM13 infusion (43). In terms of safety, AFM13 was well tolerated, with the maximum tolerated dose not reached. Most AFM13-related adverse effects were mild and resolved with supportive treatment (43).

**Figure 2:**
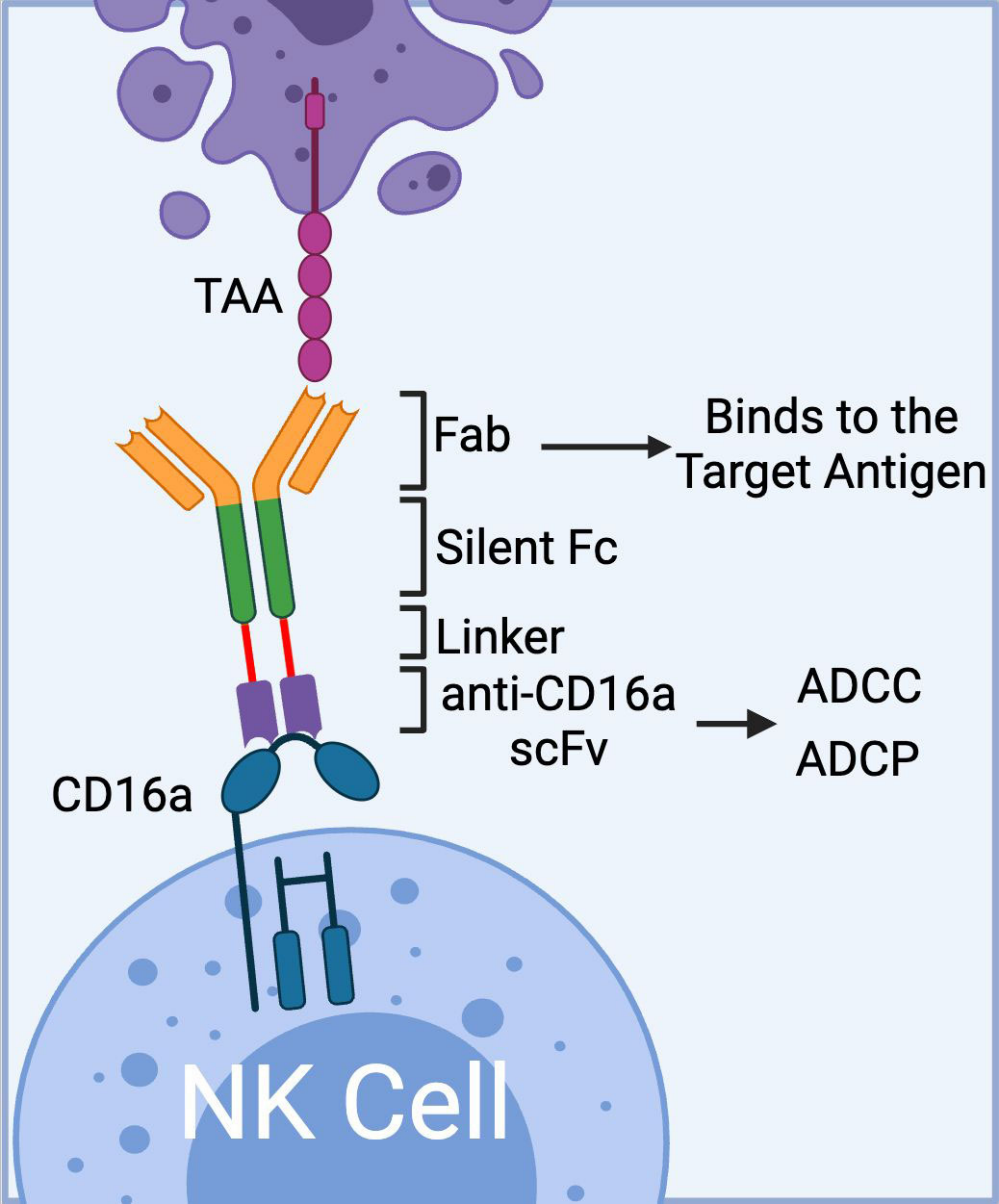
The schematics of Affimed’s ROCK^®^ platform. The platform is composed of three domains: 1) Fab region that binds to tumor associated antigen, 2) Fc region (silent) that enhances blood circulation time; and 3) bivalent anti-CD16a scFv that binds to CD16a on NK cells with high affinity mediating ADCC. In addition to ADCC, the anti-CD16a scFv could mediate ADCP via binding to CD16a receptor on macrophages. (Created with BioRender.com).

To date, Affimed has incorporated anti-CD16a scFv into various NK cell engager constructs (ROCK^®^ platform) to create Immune Cell Engagers (ICE^®^) targeting tumor-associated antigens (TAAs) such as EGFR (47), BCMA (48, 49), and CD200 (48). The ROCK^®^ platform has been shown to induce not only ADCC but also antibody-dependent cell-mediated phagocytosis (ADCP) through the activation of CD16a-expressing macrophages (**Figure 2**) (50).

In addition to AFM13, Affimed has successfully advanced another clinical trial with the AFM26 construct, targeting BCMA+ multiple myeloma cells. AFM26, which was recently licensed by Roche Inc. and rebranded as RO7297089, features an anti-CD16a scFv, P2C-47, engineered for enhanced binding affinity to both CD16a 158V and 158F allotypes (49, 51). CD16a receptor has three different allotypes based on the presence of phenylalanine (F) or valine (V) at amino acid residue 158. Each allotype (V/F, F/F, V/V, and V/F) allows the NK cells to interact with the Fc domain of mAbs with different affinities, which in turn can lead to a different clinical outcome. For more information on this subject, we would like to invite readers refer to the recent review articles by Barb et al. (2021), and Wemlinger et al. (2024) (5, 52). *In vitro* assays demonstrated that RO7297089 can effectively bind to NK cells even in the presence of high concentrations of human polyclonal IgG, mediating ADCC at a very low effector-to-target (E:T) ratio of 0.05. This led to the lysis of multiple myeloma cells with low BCMA expression, outperforming native and Fc-enhanced anti-BCMA IgGs (51).

RO7297089 has also undergone evaluation in a non-human primate (NHP) study to assess its PK profile and toxicity prior to its first-in-human clinical trial. Overall, RO7297089 exhibited a PK profile similar to that of human IgG1 antibodies (53). To correlate PK data with drug efficacy, the study quantified RO7297089’s binding to soluble BCMA or CD16a in serum and the formation of the BCMA-RO7297089-CD16a complex. These data, along with other parameters and considerations regarding interspecies differences between humans and monkeys, contributed to the initial establishment of the target-mediated drug disposition (TMDD) model for RO7297089. From this model, weekly administration of RO7297089 was recommended to ensure sustained BCMA engagement, a dosing interval that was ultimately applied in the phase 1 clinical study for multiple myeloma patients (54). In the phase 1 dose-escalation study, RO7297089 exhibited a favorable safety profile, with no maximum tolerated dose reached (54). Adverse effects during the clinical trial were manageable and did not lead to treatment discontinuation. Two cases of low-grade cytokine release syndrome induced by RO7297089 were observed, but both were resolved with supportive care. Notably, partial remission was achieved in patients receiving RO7297089 (54).

Other notable constructs from the ROCK^®^ platform include AFM24, which targets EGFR, and AFM28, which targets CD123 (47, 55). The complete list of immune cell engagers currently under development by Affimed is presented in **Table 1**.

**Table 1:** List of Affimed’s immune cell engagers in preclinical/clinical trials.

Platform Name	Effector Arm: TAA	Company	Clinical Trial	Registration#
ROCK/AFM13	CD16A:CD30	Affimed	Phase 1 (Completed)	NCT01221571
ROCK/AFM13	CD16A:CD30	University of Cologne	Phase 2 (Completed)	NCT02321592
ROCK/AFM13	CD16A:CD30	Affimed	Phase 1b/2a (Completed)	NCT03192202
ROCK/AFM13	CD16A:CD30	Affimed	Phase 2 (Completed)	NCT04101331
ROCK/AFM13+Adoptive NK Cells	CD16A:CD30	Affimed & M.D. Anderson	Phase 1/2 (Active, not recruiting)	NCT04074746
ROCK/AFM13+Pembrolizumab	CD16A:CD30	Affimed & Merck	Phase 1	NCT02665650
ROCK/AFM13+AB-101 (Allogeneic NK cells)	CD16A:CD30	Affimed	Phase 2 (Recruiting)	NCT05883449
ROCK/AFM24	CD16A:EGFR	Affimed	Phase 2 (Completed)	NCT04259450
ROCK/AFM24 + SNK01 (ADOPTIVE NK CELLS)	CD16A:EGFR	Affimed & NKGen Biotech	Phase 1 (Terminated)	NCT05099549
ROCK/AFM24 + Atezolizumab (ANTI-PD-1)	CD16A:EGFR	Affimed	Phase 1/2a (Recruiting)	NCT05109442
ROCK/AFM28	CD16A:CD123	Affimed	Phase 1 (Recruiting)	NCT05817058
ROCK/AFM26 (RO7297089)	CD16A:BCMA	Affimed + Genentech	Phase 1 (Completed)	NCT04434469
ROCK/AFVT-2101	CD16A:FOLR1	Affimed + Affivant	Pre-IND	Not Applicable

### 2.2 VHH-based NKCEs targeting CD16a receptors

In addition to scFvs, single-domain antibodies derived from the variable domain of heavy chain-only antibodies in camelids, also known as VHHs or nanobodies (56), have been employed in the development of CD16a-targeting NK cell engagers. VHHs are the smallest intact antibody fragments, derived from camelid IgG2/IgG3 antibodies, and are notable for their lack of a light chain (57). For example, Nikkhoi et al. (2022) successfully isolated an anti-CD16a VHH (clone C1) that exhibited sub-nanomolar affinity for the CD16a receptor while avoiding interaction with the inhibitory CD32b receptor on B cells or CD16b-NA1 on neutrophils (30). Utilizing the C1 VHH, a bivalent bispecific killer cell engager (BiKE) was engineered by fusing C1 with a high-affinity anti-HER2 VHH (clone E5). *In vitro* ADCC assays revealed that the E5C1 BiKE could activate NK cells and was over 100-fold more potent than trastuzumab in killing HER2+ breast and ovarian cancer cells (30). Subsequent studies demonstrated that E5C1 BiKE could not only activate CD16-expressing NK cells and macrophages *in vitro* but also eradicate HER2+ metastatic ovarian tumors from the peritoneal cavity of NK-humanized hIL-15 and hIL-2 NOG mice (58). Additionally, the E5C1 BiKE was shown to activate CD16+ THP-1-derived M1 macrophages to eliminate HER2+ ovarian cancer cells through ADCP. Later, Yang et al. (2024), utilized the aforementioned bivalent BiKE as a template and engineered a tetravalent BiKE by recombinantly fusing two anti-CD16a and two anti-HER2 VHHs in tandem. It was demonstrated that the tetravalent BiKE achieved ultra-high affinity due to avidity and high anticancer activity without mediating NK cell fratricide. This NKCE construct developed by Nikkhoi et al. has been licensed to Sauvie Inc. (CAM-V^®^ platform) for further development.

**Figure 3:**
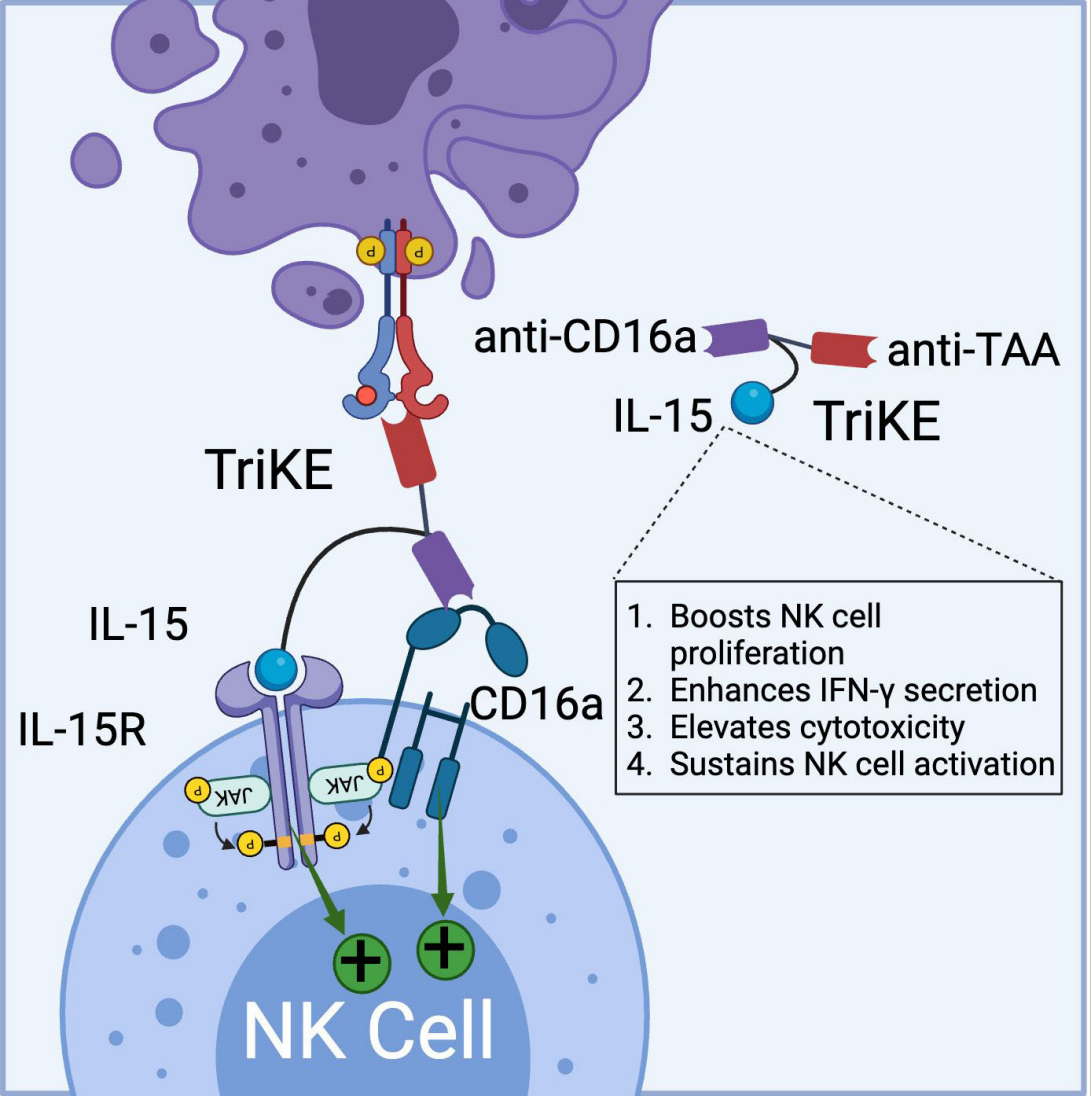
Schematic representation of TriKE composed of CD16a binding domain (VHH-based), tumor associated antigen binding domain (VHH-based), and IL-15 domain (binds to IL-15R). TriKE can bind and activate both CD16a and IL-15 signaling pathways to boost NK cell activation, proliferation and cytokine secretion. (Created with BioRender.com).

Similar to BiKEs, trispecific killer cell engagers (TriKEs) are being developed, capable of simultaneously targeting three antigens (28, 59). GT Biopharma currently has several candidates (TriKE^®^ platform) in both clinical and preclinical pipelines. The first generation of TriKEs developed by GT Biopharma utilized anti-CD16a scFv, anti-tumor-associated antigen (TAA) scFv, and interleukin-15 (IL-15) (**Figure 3**).

Targeting the IL-15 receptor is a strategic choice, as it can enhance NK cell proliferation, IFN-γ secretion, and cytotoxicity, positioning it as a promising candidate for NK-based cancer immunotherapy. Various studies have demonstrated the ability of these TriKEs to activate CD16a, induce NK cell proliferation, and increase the secretion of cytokines such as IFN-γ, GM-CSF, MIP1α, and TNF-α (60, 61). Building on this platform, GTB-3550, a first-generation TriKE candidate, entered a phase 2 clinical trial for the treatment of refractory/relapsed acute myeloid leukemia (AML) (62). Clinical trial data (NCT03214666) have shown minimal cytokine release syndrome in patients at doses ranging from 5-150 µg/kg/day. Due to its small molecular size (~60 kDa) and the absence of an Fc domain, GTB-3550 was administered daily, with a serum half-life calculated at 2.2-2.5 hours post-injection. Clinical benefits observed included a significant reduction of up to 63.7% in bone marrow plasmablast levels, proving effective in AML patients (62). Additionally, the treatment restored the patient’s endogenous NK cell function, proliferation, and immune surveillance. More recently, GT Biopharma has developed a second-generation TriKE by substituting the anti-CD16a scFv with an anti-CD16 VHH. This second-generation TriKE demonstrated superior functionality compared to the first generation, particularly in terms of binding and affinity, while maintaining a similar preclinical safety profile. Furthermore, NK cells activated by the second-generation TriKE expressed higher levels of CD107a, CD25, and CD69 compared to those activated by the first generation (63). Given the superior *in vitro* and *in vivo* results, GT Biopharma has terminated the GTB-3550 (first generation) clinical trial in favor of the GTB-3650 (second generation) trial. A comprehensive list of immune cell engagers currently under development by GT Biopharma is provided in **Table 2**.

**Table 2:** List of GT Biopharma TriKE^®^ and Sauvie’s CAM-V^®^ platforms in preclinical/clinical trials.

Platform Name	Effector Arm: TAA: Cytokine	Generation	Clinical Trial	Registration No.
TriKE/GTB-3550	CD16A:CD33:IL-15	1^st^	Phase 1/2 (Terminated)	NCT03214666
TriKE/GTB-3650	CD16A:CD33:IL-15	2^nd^	Phase 1 (Not yet recruiting)	NCT06594445
TriKE/GTB-5550	CD16A:B7H3:IL-15	2^nd^	IND-Submission	Not Applicable
TriKE/GTB-4550	CD16A:PD-L1:IL-15	2^nd^	Preclinical	Not Applicable
TriKE/GTB-6550	CD16A:HER2:IL-15	2^nd^	Preclinical	Not Applicable
TriKE/GTB-7550	CD16A:CD19:IL-15	2^nd^	Preclinical	Not Applicable
CAM-V/SV-0400	CD16A:HER2	Not Applicable	Preclinical	Not Applicable

### 2.3 NKCEs targeting NKG2D receptors

The natural killer group 2D (NKG2D) is an activating receptor that is abundantly present on all NK cells, NKT cells, and subsets of γδ T cells (64). For more detailed discussion on the role of NKG2D receptor as a regulator of cytotoxic immune cell responsiveness, we refer readers to an excellent review article by Wensveen et al. (2018) (65). To date, eight distinct ligands have been identified for NKG2D, which are classified into two groups: MHC class I-like proteins (MICA/B) and UL16 binding proteins (ULBP1-6) (66, 67). NKG2D is a type II transmembrane receptor characterized by a C-type lectin-like ectodomain that forms disulfide-bonded homodimers when binding to its ligands (66). In the cytoplasm, each NKG2D monomer associates with two DAP10 adaptor proteins, enabling the initiation of downstream signaling pathways (68). The activation of NK cells via the NKG2D receptor provides a compelling rationale for the development of NK cell engagers for cancer therapy.

For example, Chan et al. (2018) developed a BiKE that bridges CS1-expressing multiple myeloma cells and NKG2D receptors on NK cells. This BiKE successfully activated NK cells, leading to immune synapse formation, degranulation, and TNF-α secretion in IL-2-primed peripheral blood mononuclear cells (PBMCs) (69). Similarly, Raynaud et al. (2020) demonstrated that a VHH-based NKG2D-targeted BiKE could elicit effector functions in freshly isolated, resting NK cells by enhancing the tightness of the immune synapse. This increased tightness was attributed to the relatively small size of VHH-based immune cell engagers (70). Although the activation through NKG2D receptors is generally considered less robust than through CD16a receptors (37), NKG2D-targeted BiKEs offer promising alternative treatment options, particularly for patients with reduced CD16a expression (70).

**Figure 4:**
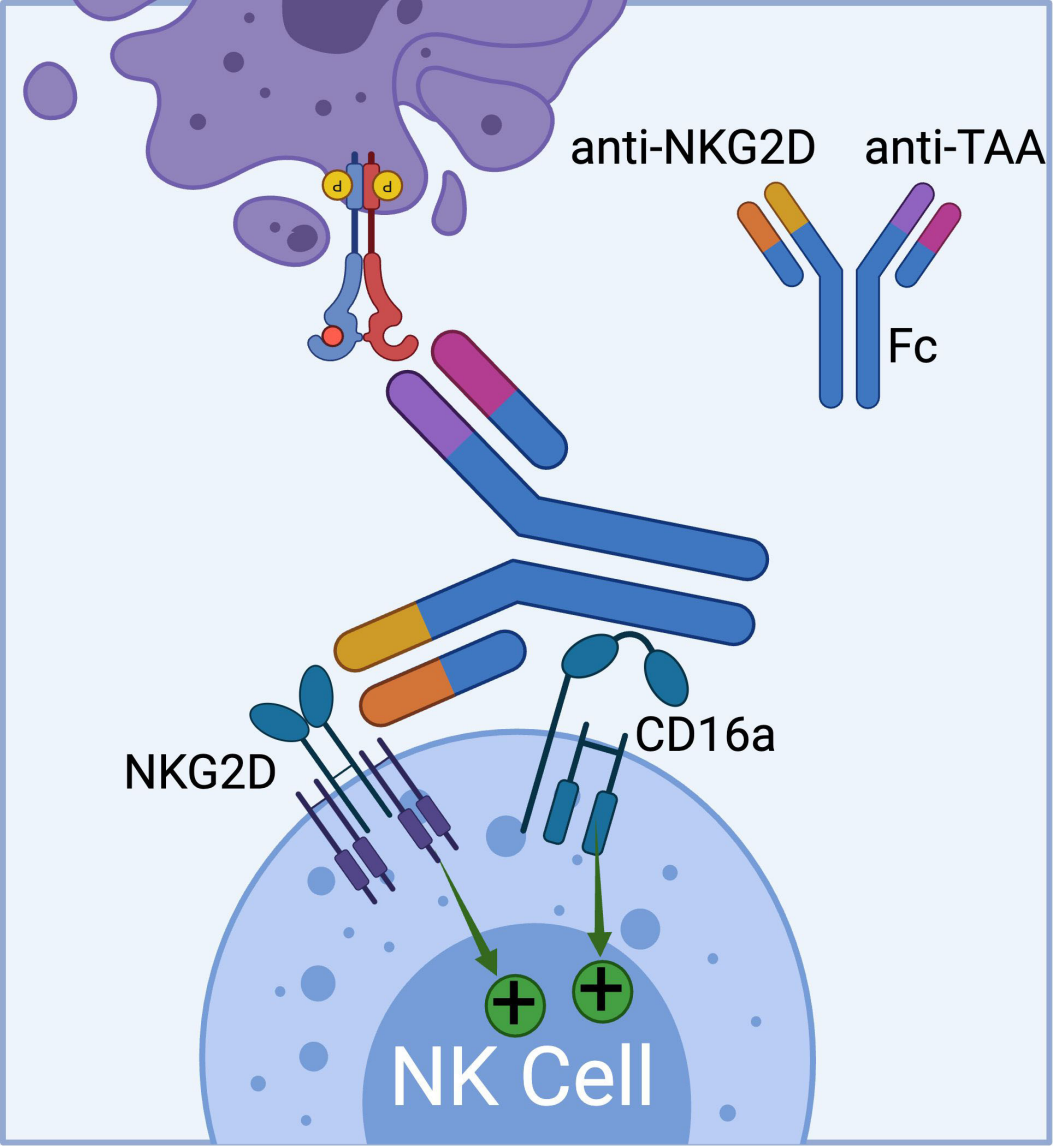
The schematics of TriNKET™ platform from Dragonfly (obtained from patent# AU2018220736A1), which can bind to both CD16a and NKG2D activating receptors on NK cells. (Created with BioRender.com).

Currently, Dragonfly Therapeutics is leveraging its TriNKETs^®^ platform for cancer immunotherapy (**Figure 4**) (71–73). NKCEs generated from TriNKET^®^ platform simultaneously engage two activating receptors, NKG2D and CD16a, on NK cells. This dual-engagement strategy enables NK cells to receive activation signals through distinct mediators. While CD16a transmits signals via CD3ζ and/or FcϵRIγ, NKG2D signals through DAP10. As a result, even if CD16 undergoes internalization or shedding, NK cells remain activated through the secondary receptor, NKG2D.

TriNKET^®^ NKCEs have been engineered to target various TAAs on cancer cells, including HER2, BCMA (72), and CD33 (73). CC-96191, also known as DF-2001, demonstrated maximum efficacy in eliminating CD33+ AML cells through the co-engagement of NKG2D and CD16a receptors. Notably, its maximal activity against AML cells was maintained even in the presence of high concentrations of the soluble NKG2D ligand, MICA (73). Dragonfly’s DF1001 candidate, which targets the HER2 receptor, has completed a Phase 1 clinical trial and reported positive outcomes in terms of safety and efficacy (NCT04143711). In terms of safety, no high-grade treatment-related adverse effects or dose-limiting toxicities were detected, and the maximum tolerated dose was not reached. A partial response was observed in 5 patients, with 22 patients achieving stable disease. In addition, the data demonstrated a reduction in tumor burden across various tumor types, particularly in heavily pre-treated patients with low HER2 expression. The overall clinical benefit rate was 39.7% (https://doi.org/10.1200/JCO.2023.41.16_suppl.2508). Dragonfly currently has several candidates in both clinical and preclinical pipelines, which are summarized in **Table 3**.

**Table 3:** List of Dragonfly’s TriNKET technology in preclinical/clinical trials.

Platform Name	Effector Arm: Tumor Antigen	Company	Clinical Trial	Clinical trial ID
TriNKET/DF1001	NKG2D/CD16:HER2	DragonFly	Phase 1/2 (Recruiting)	NCT04143711
TriNKET/DF9001	NKG2D/CD16:EGFR	DragonFly	Phase 1 (Recruiting)	NCT05597839
TriNKET/DF2001 (CC-96191)	NKG2D/CD16:CD33	DragonFly + BMS	Phase 1 (Recruiting)	NCT04789655
TriNKET/DF3001 (CC-92328)	NKG2D/CD16:BCMA	DragonFly + BMS	Phase 1 (Not Recruiting)	NCT04975399
TriNKET/DF4001	NKG2D/CD16/undisclosed	DragonFly + BMS	Preclinical	Not Applicable
TriNKET/DF5008	NKG2D/CD16:undisclosed	DragonFly + BMS	Preclinical	Not Applicable
TriNKET/DF2200	NKG2D/CD16:undisclosed	DragonFly + BMS	Preclinical	Not Applicable
TriNKET/DF8001 (MK4464)	NKG2D/CD16:undisclosed	DragonFly + Merck	Phase 1 (Active, not Recruiting)	NCT05514444
TriNKET/DF8101	NKG2D/CD16:HSPA2	DragonFly + Merck	Preclinical	Not Applicable
TriNKET/DF4101 (ABBV-303)	NKG2D/CD16:c-Met	DragonFly + AbbVie	Phase 1 (Recruiting	NCT06158958
TriNKET/DF7001	NKG2D/CD16:5T4	DragonFly + Gilead	Preclinical	Not Applicable

Despite the promising efficacy demonstrated by NKG2D-targeted NKCEs, several challenges limit their effectiveness (74). One major obstacle is the ability of cancer cells to evade immune detection by shedding NKG2D ligands (e.g., MICA/B) through protease-mediated cleavage (75). The metalloproteinase ADAM10 plays a crucial role in this mechanism by cleaving these ligands under both normal and stress conditions in various cancer types (76). By shedding NKG2D ligands into the bloodstream, cancer cells prevent the immune cells from triggering an immune response, effectively bypassing NKG2D-mediated natural immune surveillance. It has been shown that soluble NKG2D ligands, primarily MICA, can bind to NKG2D receptors on NK cells resulting in their downregulation through endocytosis and degradation impairing their cytotoxic effects (74, 77, 78). For more information related to NKG2D ligand shedding and immune evasion of cancer cells, we refer the readers to the articles by Raffaghello et al. (2004) and Fantini et al. (2023) (74, 79). Additionally, tumor-derived cytokines, such as transforming growth factor beta (TGF-β), can downmodulate NKG2D expression on NK cells (80). Reduced NKG2D receptor expression has also been observed in tumor infiltrating NK cells isolated from renal cell carcinoma patients (81), which could further complicate the effectiveness of NKG2D-targeted NKCEs.

### 2.4 NKCEs targeting NKG2C receptors

NKG2C is another important activating receptor expressed on NK cells. Upon activation, the NKG2C receptor forms a heterodimer with CD94 and signals through the DAP12 adaptor protein (82). While NKG2C expression on NK cells is typically low, it can be significantly upregulated in response to cytomegalovirus (CMV) infection (83). The elevated surface density of NKG2C offers two key advantages: it enhances NK cell cytotoxicity and competes with the inhibitory receptor NKG2A for CD94 binding, thereby helping to sustain NK cell activation (84) (85). These findings have spurred efforts to develop NKCEs that trigger NK cell effector functions via NKG2C. For instance, Chiu et al. (2021) recently reported the development of an NKG2C-targeted NKCE that bridges NKG2C on NK cells with CD33 on target cells. This interaction successfully triggered NK cell degranulation, IFN-γ secretion, and lysis of the target cells (86). Remarkably, the engineered NKG2C-NKCE was as effective as a CD16-NKCE in controlling tumor formation in mice, even though NKG2C expression on NKG2C-positive adoptive NK cells was much lower than that of CD16 (86).

### 2.5 NKCEs targeting NKp46 receptors

NKp46 is a natural cytotoxicity receptor on NK cells, with its expression highly conserved across mammalian species, including humans, mice, and monkeys (87, 88). Like CD16a, NKp46 can associate with either CD3ζ or FcRγ to transduce activating signals via the ITAM motifs within NK cells (89). In humans, NKp46 is expressed on the family of innate immune cells and is considered a marker for mature NK cells (90). Activation through NKp46 can induce cytokine production and calcium ion influx, leading to NK cell degranulation and cytolytic activity (37, 90). Notably, unlike CD16a, NKp46 maintains sustained expression on NK cells that have infiltrated tumors, without significant downregulation (91). This unique characteristic has made NKp46 an attractive target for developing NKCEs. For example, Lipinski and colleagues isolated a variety of anti-NKp46 VHHs with differing affinities via yeast surface display (92). To develop a BiKE that targets both NKp46 and EGFR, the Fab region of cetuximab (an anti-EGFR monoclonal antibody) was fused to an anti-NKp46 VHH. This BiKE effectively mediated NK cell activation and later displayed enhanced EGFR-specific tumor cell lysis compared to FDA approved EGFR-targeted antibody cetuximab (92). Gauthier et al. (2019) also reported the structure of a NKCE targeting NKp46 (91). They also included an Fc domain in the NKCE structure to enhance the NK cell activation via the CD16a receptor. They observed a synergistic activity between CD16a and NKp46 receptor which was consistent with the reports from other groups (37). Based on this discovery, Innate Pharma developed a platform called Antibody-based NK cell Engager Therapeutics (ANKET™), while Cytovia Therapeutics developed FLEX-NK platform.

**Figure 5:**
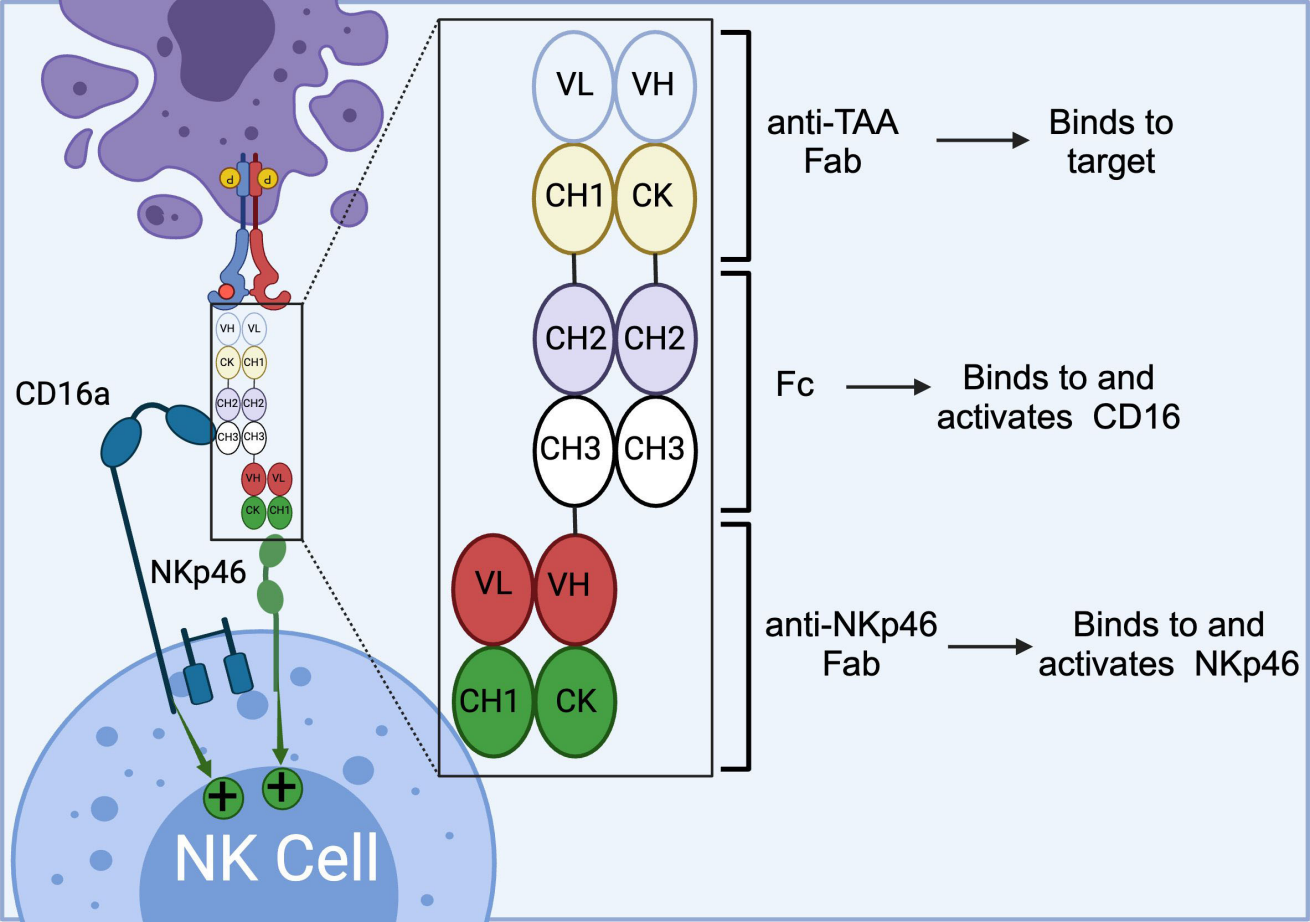
Schematics of ANKET™ technology. This NKCE, utilizes an anti-NKp46 Fab to activate NK cells via NKp46 receptor. It also boosts the ADCC via its Fc domain that engages CD16a receptor. (Created with BioRender.com).

The ANKET™ platform is engineered through fusion of an anti-NKp46 Fab to an anti-TAA Fab through IgG Fc domain, which interacts with CD16a (**Figure 5**) (91). Preclinical data indicate that ANKET™ constructs can elicit robust NK cell effector functions, including specific target cell lysis and cytokine production. Furthermore, CD20-redirected NKCEs developed using this platform demonstrated superior efficacy compared to the FDA-approved anti-CD20 monoclonal antibody obinutuzumab, both *in vitro* and *in vivo* (93). Innate Pharma currently has several ANKET™-based candidates in its development pipeline. IPH6101/SAR’579 is currently undergoing a phase 1/2 clinical study in patients with relapsed or refractory AML, B-cell acute lymphoblastic leukemia (ALL), or high-risk myelodysplasia (HR-MDS). Results from the clinical study showed that IPH6101/SAR’579 has a favorable safety profile, with no dose-limiting toxicities that would necessitate treatment termination. Encouragingly, complete remission was observed in 5 out of 15 patients (https://doi.org/10.1182/blood-2023-173162). A summary of NKp46-targeted immune cell engagers can be found in **Table 4**.

**Table 4:** The list of NKCEs that target NKp46 receptor on NK cells in preclinical/clinical studies.

Platform Name	Effector Arm: TAA	Company	Clinical Trial	Registration #	Ref.
ANKET/IPH6101/SAR’579 (SAR443579) alone	NKp46/CD16a:CD123	Innate Pharma Partnered with Sanofi	Phase 1/2 (Recruiting)	NCT05086315	Not Applicable
ANKET/IPH6101/SAR’579 combined with venetoclax and azacitidine	NKp46/CD16a:CD123	Innate Pharma Partnered with Sanofi	Phase 1/2 (Recruiting)	NCT06508489	Not Applicable
ANKET/IPH6401/SAR445514	NKp46/CD16a:BCMA	Innate Pharma Partnered with Sanofi	Phase 1/2 (Recruiting)	NCT05839626	
ANKET/IPH62	NKp46/CD16a:B7-H3	Innate Pharma Partnered with Sanofi	Preclinical	Not Applicable	
ANKET/IPH67	undisclosed	Innate Pharma Partnered with Sanofi	Preclinical	Not Applicable	
ANKET/IPH6501	NKp46/CD16a:CD20+IL-2v	Innate Pharma	Phase 1/2	NCT06088654	(93)
ANKET/NKCE	NKp46/CD19	Innate Pharma	Preclinical	Not Applicable	(91)
ANKET/NKCE	NKp46/CD20	Innate Pharma	Preclinical	Not Applicable	
FLEX-NK/CYT-338	NKp46:CD123	Cytovia Therapeutics	Preclinical	Not Applicable	(94)
FLEX-NK/CYT-303	NKp46:GPC3	Cytovia Therapeutics	Preclinical	Not Applicable	(95, 96)
VHH-based NKCE	NKp46/EGFR	Merck Healthcare KgaA	Preclinical	Not Applicable	(92)

Similar to Innate Pharma’s ANKET™ platform, Cytovia Therapeutics has leveraged its FLEX-NK platform to engineer CYT-303 and CYT-338 NKCEs, targeting the CD38 antigen for hematological malignancies and GPC-3 for hepatocellular carcinoma (95, 97). The CD38-targeted construct successfully restored the functionality of dysfunctional NK cells, resulting in the elimination of CD38+ multiple myeloma cells and significant tumor growth inhibition in mouse models (97). Additionally, this construct prevented NK cell fratricide, a common issue with anti-CD38 mAbs, such as daratumumab, by selectively activating NKp46 signaling (**Table 4**) (97).

### 2.6 NKCEs targeting NKp30 receptors

NKp30 is another pivotal natural cytotoxicity receptor that is constitutively expressed on NK cells. Upon binding to its ligands, B7-H6 or BAT3, NKp30 associates with the intracellular adaptor complex composed of CD3ζ and FcRγ, which transmits activating signals through ITAM domains (37). To date, six isoforms of the NKp30 receptor have been identified, with isoforms NKp30a and NKp30b being the most potent in inducing NK cell activation and cytolytic activities (89, 98). In patients with gastrointestinal stromal tumors, the expression levels of these NKp30 receptor isoforms on the NK cells have been shown to correlate with prognosis and survival outcomes (99, 100). In a study by Pekar et al. (2021), the potential of targeting the NKp30 receptor for the development of NK cell engagers (NKCEs) was demonstrated (101). As a first step, a panel of affinity-matured ΔB7-H6 ligands was isolated to efficiently target NKp30. These optimized ΔB7-H6 fragments exhibited up to a 45-fold higher binding affinity to NKp30 compared to the wild-type B7-H6. Subsequently, a bispecific antibody, termed an immunoligand, was engineered by fusing the affinity-matured ΔB7-H6 to the EGFR-binding Fab region derived from cetuximab. This engineered BiKE was shown to effectively activate NK cells and significantly enhance EGFR-specific tumor cell lysis compared to a BiKE constructed with wild-type B7-H6. The increased production of IFN-γ and TNF-α was also observed with the aforementioned immunoligands. In an interesting study by Klausz et al. (2022), a panel of anti-NKp30 VHHs with a wide range of binding affinities and epitope coverage for NKp30 receptors was isolated and used to create a series of EGFR-targeted NKCEs (102). All NKCEs generated in the study demonstrated the ability to eliminate EGFR-expressing cancer cells; however, the potency and efficacy of tumor cell killing varied significantly depending on the specific epitopes on NKp30 that each NKCE targeted. Importantly, the generated NKCEs were designed to bind to NKp30 receptor epitopes different from those recognized by natural ligands B7-H6. This approach allowed the NKCEs to circumvent the inhibitory effects of soluble B7-H6, which is commonly found in the serum of cancer patients (102). In a subsequent study by Boje et al. (2024), single-domain antibodies (sdAbs) were utilized to construct NKCEs targeting NKp30 to redirect NK cell cytotoxicity toward EGFR-expressing tumor cells (103). This study investigated the impact of several crucial parameters, including sdAb location, binding valencies, the targeted epitope on NKp30, and the overall antibody architecture, on the redirection capacity of NKCEs. Two NKp30-specific sdAbs were constructed: one targeting a similar epitope on NKp30 as its natural ligand B7-H6, and the other binding a non-competing epitope. For targeting EGFR-positive tumors, humanized antigen-binding domains of the therapeutic antibody cetuximab were employed. The results demonstrated that NKCEs that bivalently target both EGFR and NKp30 were superior to monovalent NKCEs in promoting NK cell-mediated tumor cell lysis. Importantly, the location and orientation of NKp30-targeting sdAbs within the NKCE sequence were found to be critical factors influencing the ultimate efficacy of NKCEs.

Given that both NKp46 and NKp30 serve as co-stimulatory activating receptors, a pertinent question arises: which receptor generates a more potent response when combined with an anti-CD16a engager? Colomar-Carando et al. conducted an intriguing study to address this question, comparing the efficacy of NKCEs that co-engage CD16a with either NKp30 or NKp46 in eliciting NK cell effector functions (104). The researchers generated TriKEs capable of targeting NK activating receptors CD16a-NKp30 or CD16a-NKp46, alongside CD19 or CD20 tumor-associated antigens (TAAs). These TriKEs were then evaluated for their ability to kill acute lymphoblastic leukemia cells expressing either CD19 or CD20. The study revealed that all NKCEs efficiently killed the NK cell-resistant MHH-CALL-4 cells, as evidenced by increased NK cell activity, including degranulation and IFN-γ production. Moreover, the CD19-targeting TriKEs were effective against primary BCP-ALL cells, even in patients who had undergone transplantation. Interestingly, the NKp30-CD16a and NKp46-CD16a NKCEs demonstrated equivalent capabilities in eliminating CD19 and CD20-expressing target cells, suggesting that NKp30 and NKp46 have comparable potency in activating NK cells (104). This observation could be attributed to the fact that the intracellular domains of both receptors are very similar, consisting of short intracellular fragments without intrinsic signaling domains, necessitating engagement with either CD3ζ or FcϵRIγ (89). A summary of immune cell engagers targeting NKp30 is provided in **Table 5**.

**Table 5:** List of NKCEs targeting NKp30 in preclinical studies.

NKCE	Effector Arm: TAA	Company	Clinical Trial	Registration #	Ref.
Ligand-based NKCE	NKp30:EGFR	Merck Healthcare KgaA	Preclinical	Not Applicable	(101)
VHH-based NKCE	NKp30:EGFR	Merck Healthcare KgaA	Preclinical	Not Applicable	(105)
NKCE	NKp30:CD19 or CD20	Colomar-Carando et al	Preclinical	Not Applicable	(104)

## 3 Strategies to improve NKCE efficacy

NKCEs rely on the functionality of NK cells to achieve their intended therapeutic effects. However, it is common for NK cells in cancer patients to be dysfunctional or inept in recognizing tumor cells, presenting a significant challenge for effective treatment. To address this issue, various strategies have been employed to restore NK cell functionality and enhance the efficacy of NKCEs.

### 3.1 Targeting Siglec-7 (CD328) inhibitory receptors

Sialic acid-binding immunoglobulin-like lectin 7 (Siglec-7) has recently emerged as a promising target in cancer immunotherapy. Siglec-7 is primarily expressed on monocytes and NK cells, where it functions as an inhibitory receptor, playing a critical role in the regulation of NK cell activity (106, 107). Siglec-7 recognizes sialic acid residues present on sialylated glycoconjugates as its ligands and operates in a sialic acid-dependent manner (108). Upon binding to sialic acid, Siglec-7 transmits inhibitory signals through an immunoreceptor tyrosine-based inhibitory motif (ITIM) within NK cells (107).

Many tumors have been reported to exhibit significant levels of surface sialylation, which allows them to engage Siglec-7 and evade NK cell-mediated immunosurveillance (109, 110). Consequently, blocking the interaction between Siglec-7 and its ligands on cancer cells has been proposed as a viable strategy to enhance NK cell-mediated cytotoxicity (87). For instance, Bordoloi et al. (2023) explored the potential of this strategy by engineering a NKCE aimed at targeting ovarian cancer cells. The team constructed a BiKE that could simultaneously block Siglec-7 on NK cells and target the follicle-stimulating hormone receptor (FSHR) on ovarian cancer cells (111). This BiKE was demonstrated to effectively inhibit the negative signaling mediated by Siglec-7, thereby leading to NK cell activation and the subsequent elimination of ovarian cancer cells in both *in vitro* and *in vivo* models (112). These findings underscore the therapeutic potential of Siglec-7 blockade as a means to maintain NK cells in an activated state when they encounter target cells, ultimately enhancing their cytotoxic efficacy against cancer.

### 3.2 Inclusion of Fc domain in NCKE structure to exploit CDC and ADCP

An effective strategy to enhance the therapeutic activity of NK cell engagers (NKCEs) involves incorporating a functional Fc domain to leverage both complement-dependent cytotoxicity (CDC) and ADCP mechanisms. The CH2 domains within these functional Fc regions enable NKCEs to recruit complement proteins, initiating the classical CDC pathway. When an NKCE binds to a tumor cell, complement protein C1q first binds to the NKCE’s Fc domain, triggering a cascade that recruits additional complement proteins, including C4, C2, C3b, C5b, C6, C7, C8, and C9 (113). These complement complexes then assemble into membrane attack complexes, creating pores in the tumor cell membrane that ultimately lead to cell lysis (114). For instance, BL-01, a tetravalent Fc-bearing NKCE, has been demonstrated to efficiently induce CDC against CD20+ B-cell lymphoma cells (115). Similarly, cross-over dual-variable Ig-like proteins (CODV-Ig) with functional Fc domains have shown the ability to recruit complement proteins, effectively eliminating target cells via CDC mechanisms (116). NKCEs such as ISB1442 (117), CYT-338 (94), and CYT-303 (95) have also been reported to effectively induce CDC against CD38+ lymphoma cells and GPC-3+ hepatocellular carcinoma cells. Beyond the complement cascade, the CH2 domain within the Fc region can bind to Fcγ receptors on macrophages, stimulating them to engage in ADCP (118). In this process, activated macrophages engulf the opsonized cancer cells, degrading them within phagosomes through acidification (119). This ADCP-mediated cell elimination has been observed for CYT-338 and CYT-303 as previously discussed.

### 3.3 Inclusion of cytokines into NKCEs

In addition to complement recruitment, cytokines have been employed to further improve the efficacy of NKCEs. Beyond IL-15, which was previously discussed, other cytokines such as IL-2 have been explored for their potential to enhance NK cell activity. A variant of interleukin-2 (IL-2v) was incorporated into a tetrafunctional NKCE, known as IPH6501 (93). Notably, this IL-2 variant does not bind to the α-subunit of IL-2 receptor, thereby limiting the activation of regulatory T cells and reducing the risk of IL-2-mediated toxicity (93). Given the potent stimulatory effects of IL-2, IPH6501 has been shown to induce the proliferation and cytolytic activity of primary human NK cells, as well as the secretion of cytokines and chemokines in both *in vitro* and *in vivo* models of B cell malignancies (93).

### 3.4 Utilization of alternative NK cell sources

When NK cells from patients are insufficiently functional to support NKCE activity, alternative functional NK cell sources have been investigated. These sources include non-engineered allogeneic NK cells from healthy donors and iPSC-derived NK cells. For instance, AB-101, developed by Artiva Biotherapeutics, is an expandable, non-engineered allogeneic NK cell source isolated from the umbilical cord blood (UCB) of healthy donors. Most AB-101 NK cells express high-affinity CD16a (158 V/V) and other activating receptors at significant levels. In combination with monoclonal antibodies (mAbs), AB-101 has been shown to effectively induce ADCC and inhibit tumor growth *in vivo* (120). Moreover, AB-101 has demonstrated efficacy when combined with AFM-13 (Affimed), an NKCE targeting CD30+ T cell lymphoma. This combination therapy is currently being evaluated in a phase 2 clinical trial (NCT05883449) (121). However, a limitation of using NK cells from different donors is the potential for batch-to-batch variations and heterogeneity of the cell source, which may affect the reproducibility of therapeutic outcomes.

iPSC-derived NK cells offer an alternative with significant advantages, including homogeneity, engineerability, and an unlimited lifespan, minimizing batch-to-batch variations. Compared to NK cells isolated from UCB, iPSC-derived NK cells can be further engineered to enhance NKCE activity. For example, Chiu et al. (2021) engineered iPSC-derived NK cells to express NKG2C receptors and adaptor signaling molecules to improve the therapeutic efficacy of NKG2C-targeted NKCEs (86). Additionally, iPSC-derived NK cells can be modified to express non-cleavable high-affinity CD16a receptors, further promoting engagement with NKCEs and enhancing NK cell activity and proliferation (122). Fate Therapeutics is currently one of the leading companies in utilizing iPSC-derived NK cells for cancer therapy.

In a similar approach, Cytovia Therapeutics has utilized TALEN^®^-based gene-editing technology to modify iPSCs, inserting genes that enhance NK cell effector functions and deleting those that impair them. By leveraging the unlimited proliferative capacity of iPSCs, a selected single iPSC clone was expanded and differentiated into NK cells, referred to as iNK cells. As highlighted on their website, Cytovia Therapeutics is currently evaluating the efficacy and safety of iNK cells in combination with their FLEX-NK platform in preclinical studies for the treatment of multiple myeloma, hepatocellular carcinoma, and other cancers.

### 3.5 Targeting dual antigens to prevent tumor escape

While NKCEs have demonstrated their ability to effectively eliminate tumors, cancer cells may still evade immunosurveillance by introducing mutation to the NK cell binding site or downregulating TAAs. To counter these escape mechanisms, NKCEs have been designed to concurrently target two TAAs. These dual-targeting NKCEs have shown enhanced anti-tumor activities and the ability to block inhibitory signaling pathways (111, 123). For instance, Steinmetz et al. (2016) reported the development of a cross-over dual-variable Ig-like protein (CODV-Ig) platform, which features a circular, self-contained structure that maintains the parental antibody affinities for two antigens without positional effects (116). CODV-Ig was engineered to target both HER2 and HER3 antigens on the cell surface, effectively blocking their heterodimerization. This not only resulted in the blockade of mitogenic signaling but also in ADCC-mediated killing of cancer cells (116). Other examples of dual TAA-targeting NKCEs include those targeting EGFR/PD-L1 (59, 124, 125) and CD47/CD38 (117, 126), among others.

### 3.6 Optimizing the NKCE’s half-life

The half-life and systemic exposure of NKCEs are critical determinants of their therapeutic efficacy. Typically, the half-life of NKCEs is influenced by their molecular weight and sequence. Structurally, NKCEs can be categorized into two groups: those containing an Fc fragment and those without it. The PK profile of NKCEs that incorporate an Fc fragment is often influenced by the “sink effect.”. This phenomenon arises from the non-specific binding of the Fc fragment within the NKCE sequence to various activating and inhibitory Fcγ receptors on immune cells, such as CD16b on neutrophils or CD32b on B cells (50). In contrast, NKCEs engineered to selectively bind Fcγ receptors, particularly CD16a on NK cells, exhibit reduced non-specific binding and, consequently, a lower susceptibility to the sink effect. Similar to the Fc fragment, the antigen-binding domain of NKCEs can interact with soluble target antigens present in plasma, potentially altering the PK profile. These soluble antigens often result from the shedding of extracellular domains of membrane-bound receptors, including CD16b on neutrophils or tumor-associated antigens (TAAs) (127–129). Elevated levels of soluble target antigens are frequently observed in the serum of cancer patients. For instance, the concentration of soluble BCMA is significantly higher in the serum of multiple myeloma patients compared to healthy individuals, creating an “antigen sink” that impacts NKCE pharmacokinetics (128, 130). Studies have demonstrated an inverse relationship between the concentration of soluble antigens and both drug exposure and therapeutic efficacy in clinical settings (131, 132). Additionally, shedding of extracellular domains to generate soluble antigens is recognized as a major cancer drug resistance mechanism. To counteract the sink effect associated with soluble antigens, optimizing dosage and dosing intervals is essential (53, 54). Beyond tumor-derived soluble antigens, the expression of target antigens on healthy tissues can further complicate the sink effect, as their distribution across the body influences NKCE biodistribution (127). Although no studies have specifically examined NKCE disposition related to target antigens on healthy tissues, insights from T cell engager studies suggest potential strategies. For instance, NKCE tumor selectivity may be enhanced by targeting multiple co-expressed tumor antigens or increasing the binding valency to tumor-specific antigens. Overall, research into the sink effect on NKCEs remains limited, underscoring the need for further studies to address this challenge effectively.

In addition to the sink effect, NKCEs containing Fc fragments exhibit an extended half-life due to their relatively large molecular size and their capacity for neonatal Fc receptor (FcRn)-mediated recycling. FcRn plays a critical role in regulating the half-life and recycling of IgG and albumin, contributing to prolonged antibody persistence within the body (133, 134). Expressed across a variety of cells, including endothelial and epithelial cells, antigen-presenting cells, and even within tumor microenvironments, FcRn influences antibody distribution and therapeutic outcomes. At acidic pH, FcRn binds IgG with high affinity, facilitating recycling and transcytosis, while at physiological pH, it releases the antibody. Within tumors, FcRn expression can further impact therapeutic antibody delivery and efficacy, enhancing antigen presentation and immune activation through sustained exposure to IgG-bound antigens (135, 136). For instance, NKCEs with an IgG-like format, developed using Affimed’s ROCK^®^ platform, demonstrate the longest serum half-life, followed by NKCEs with an Fc-fusion structure (50). Conversely, NKCEs derived from the ROCK^®^ platform that consist solely of scFvs and lack the Fc domain display a markedly shorter half-life, sometimes as brief as 18 hours (50). This reduced half-life is attributed to the absence of FcRn-mediated recycling and the rapid renal clearance associated with their smaller molecular weight. To address this limitation, NKCEs with shorter half-lives can be engineered by fusing them with a high-affinity anti-human serum albumin (HSA) modality, thereby extending their half-life and improving systemic exposure (137). The NKCEs with longer half-life require less dosing frequency and are more likely to generate favorable therapeutic efficacy. However, it is believed that NKCEs with potent cytotoxic profiles may benefit from a shorter half-life to minimize the risk of acute adverse effects (50). Consequently, PK modulation of NKCEs through half-life adjustment must be carefully considered on a case-by-case basis to balance efficacy and safety.

Beyond molecular structure and weight, the immunogenicity of NKCEs also plays a pivotal role in determining their half-life and PK profile. NKCEs that incorporate moieties derived from non-human species, such as murine (scFvs) or camelid (VHHs), may exhibit immunogenicity, leading to the generation of anti-drug antibodies (ADAs). The presence of ADAs can result in rapid NKCE clearance and reduced systemic exposure. The mechanisms underlying ADA generation against monoclonal antibodies (mAbs) are explored in depth in a comprehensive review by Vaisman-Mentesh (138). Briefly, ADAs can be produced via T cell-dependent or T cell-independent B cell activation pathways. In the T cell-independent mechanism, therapeutic proteins cross-react with B cell receptors (BCRs) on B cells, stimulating them to differentiate into plasma cells that produce ADAs. ADAs generated through this mechanism are predominantly low-affinity IgM antibodies (139). In contrast, the T cell-dependent mechanism involves antigen-presenting cells, primarily dendritic cells, and T lymphocytes. Dendritic cells recognize therapeutic proteins as foreign antigens, process them, and present them in complex with MHC class II molecules to CD4+ helper T cells. These T cells then interact with B cells, inducing their transformation into plasma cells, which subsequently secrete high-affinity IgG ADAs (139).

To mitigate immunogenicity concerns, humanization of NKCE components is a viable strategy, particularly for scFv-based NKCEs derived from murine sources. Generally, VHH-based NKCEs exhibit low immunogenicity, owing to their small size and the high degree of homology (75-90%) between their encoding genes and those of the VH3 family of human antibodies (140–142). If an immunogenic response is observed, VHHs can be humanized using established strategies designed to reduce their immunogenic potential (143–145). However, it is important to recognize that humanization does not inherently guarantee reduced immunogenicity, as many humanized mAbs have still induced ADA formation in clinical settings. Therefore, efforts to enhance NKCEs’ systemic exposure by minimizing immunogenicity should be carefully planned and rigorously evaluated using various *in vitro* assays to predict the immunogenicity of biologics. For instance, assessing the activation and proliferation of CD4+ helper T cells, which play a crucial role in the T cell-dependent B cell activation pathway, after exposure to biotherapeutics, can provide valuable insights (146). Additionally, T-cell and B-cell epitope mapping can be employed to evaluate the immunogenic potential of therapeutic biologics. These approaches are integral to predicting and managing immunogenicity, ultimately ensuring the safe and effective use of NKCEs in clinical practice (139).

## 4 Conclusions and perspectives

In conclusion, NK cell engagers (NKCEs) have demonstrated promising efficacy and safety in both preclinical and clinical studies. Unlike conventional monoclonal antibodies (mAbs), NKCEs can be engineered to precisely target a broad range of NK cell-activating receptors and multiple tumor-associated antigens (TAAs) within a single construct, significantly enhancing their anticancer activity. By leveraging protein recombination technology, NKCEs can also be designed to incorporate various cytokines and chemokines that enhance NK cell functionality, thereby optimizing therapeutic efficacy. Moreover, compared to the complex manufacturing processes and high costs associated with chimeric antigen receptor NK (CAR-NK) cell therapy, NKCEs offer a more cost-effective alternative, making them accessible to a larger patient population. Importantly, NKCEs possess the unique ability to simultaneously target multiple TAAs using simple protein engineering techniques. This capability holds great promise for addressing challenges associated with tumor drug resistance, antigen heterogeneity, and tumor immune escape mechanisms. However, several challenges must be addressed to fully realize the therapeutic potential of NKCEs. The functionality of NK cells is paramount to the success of NKCE therapy, yet the reduced expression of activating receptors such as CD16 and NKG2D and enhanced expression of inhibitory checkpoint receptors such as LAG-3, NKG2A, and TIGIT are frequently observed in the NK cells of cancer patients (147, 148). Additionally, the immunosuppressive tumor microenvironment (TME) of solid tumors can further compromise the proliferation and functionality of NK cells. For instance, transforming growth factor-beta (TGF-β) present in the TME of solid tumors is known to downregulate the expression of granzyme B and perforin, significantly inhibiting the cytolytic activity of NK cells (149, 150). To overcome these barriers, the inclusion of functional NK cells as part of NKCE-based treatments is essential to ensure the anticipated therapeutic outcomes. The source of functional NK cells could be ex vivo expanded allogenic, autologous, or iPSC-derived. In the context of solid tumors, combining NKCEs with strategies that block immunosuppressive cytokines may help restore NK cell effector functions. Furthermore, combining NKCEs with other treatment modalities may lead to synergistic effects in cancer therapy. For example, the combination of chemotherapy with immunotherapy could be particularly beneficial in the treatment of solid tumors (151, 152). Chemotherapy not only eliminates a significant portion of cancer cells but also enhances tumor perfusion, facilitating the infiltration of immune cells into the tumor mass. Notably, mild chemotherapy has been shown to upregulate NKG2D ligands on cancer cells while preserving immune cells, thereby enhancing the efficiency of the immune response against cancer cells (53, 152). In addition to chemotherapy, combining NK cell engagers (NKCEs) with CAR-NK cell therapy presents a promising strategy with potential synergistic benefits. This dual approach harnesses CAR-NK cells as a source of highly functional NK cells, while NKCEs—particularly those with incorporated cytokine modules—promote CAR-NK cell proliferation and enhance their persistence in patients. This synergy can lead to improved anti-tumor efficacy. Furthermore, by allowing NKCEs and CAR-NK cells to target multiple TAAs, this combination minimizes the risk of tumor escape, potentially resulting in more durable therapeutic outcomes. In summary, NKCEs represent a versatile and potent class of therapeutic molecules that not only activate NK cells but also address the limitations of conventional monoclonal antibodies (mAbs) and CAR-based immunotherapies. NKCE, whether used alone or in combination with autologous NK cells, have shown limited effectiveness in some clinical trials, particularly when administered to heavily pre-treated cancer patients (e.g., NCT05099549, https://doi.org/10.1200/GO.2023.9.Supplement_1.26). These patients frequently undergo extensive chemotherapy, which can deplete NK cells and lead to lymphopenia, resulting in an immune system compromised by a reduced population of functional immune cells. In such immunocompromised conditions, NKCE therapies face challenges, as the weakened environment cannot fully support NK cells’ intended cytotoxic action against cancer cells. This underscores the importance of careful patient selection and timing of treatment to maximize the therapeutic potential of NKCE-based therapies. Published clinical data suggest a promising approach that combines NKCEs with allogeneic NK cells, enhancing immune responses against cancer cells and potentially improving outcomes for patients resistant to conventional treatments. This combination strategy leverages the advantages of NK cells, which naturally present a minimal risk of causing graft-versus-host disease (GvHD), a significant concern with T-cell therapies. Unlike T cells, NK cells do not require genetic modification to avoid GvHD, making them a safer and more feasible option for allogeneic applications. This characteristic allows NK cell therapies to be more broadly accessible, particularly benefiting patients who may not be suitable candidates for autologous immune cell treatments. Moreover, advancements in NKCE design have led to the development of CD16a variants resistant to shedding. Typically, activated NK cells upregulate the enzyme ADAM17, which mediates the shedding of CD16a to regulate immune responses. However, using CD16a-resistant variants in NKCEs helps maintain their efficacy, even in environments with high ADAM17 activity, thereby enhancing the persistence of NK cell engagement and cytotoxicity. Furthermore, Combining NKCEs with CAR-T cells offers a synergistic approach to cancer therapy by harnessing both innate and adaptive immune responses. This dual-cell strategy could provide a more robust and comprehensive immune assault on cancer cells, leveraging both the unique targeting abilities of CAR-T cells and the natural cytotoxicity of NK cells. One of the primary challenges in cancer immunotherapy is tumor heterogeneity, which can lead to recurrence, especially in solid tumors. CAR-T cells are highly target-driven, directly recognizing and eliminating cancer cells that express specific antigens. However, this specificity can be a limitation, as tumors may evade detection by downregulating target antigens or through antigen loss, resulting in relapse. In contrast, NK cells possess a multitude of innate activating receptors, enabling them to recognize and kill a wide range of cancer cells, including those that may escape CAR-T cell targeting. As the field continues to evolve and challenges are steadily addressed, NKCEs hold great promise as an alternative or complementary approach to existing cancer immunotherapies, potentially offering more effective and accessible treatment options for patients.
